# Dental caries in 12-year-old Brazilian adolescents: a comparative analysis of the last Three National Surveys

**DOI:** 10.1590/1807-3107bor-2025.vol39.0047

**Published:** 2025-05-19

**Authors:** Viviane Elisângela GOMES, Mara VASCONCELOS, Milena Ribeiro GOMES, Andreia Maria Araújo DRUMMOND, Rosa Núbia Vieira de MOURA, Rafaela da Silveira PINTO, Renato Taqueo Placeres ISHIGAME, Joana Danielle Brandão CARNEIRO, Raquel Conceição FERREIRA

**Affiliations:** (a) Universidade Federal de Minas Gerais – UFMG, School of Dentistry, Department of Social and Preventive Dentistry, Belo Horizonte, MG, Brazil.; (b) Universidade Federal de Minas Gerais – UFMG, School of Dentistry, Graduate Program in Dentistry, Collective Health, Belo Horizonte, MG, Brazil.; (c) Ministério da Saúde, National Oral Health Coordination, Brasília, DF, Brazil.

**Keywords:** Oral Health, Dental Caries, Adolescent, Health Surveys

## Abstract

This study aimed to compare caries experience, prevalence of caries-free and untreated caries in 12-year-old adolescents, based on data from the national epidemiological surveys of 2003, 2010, and 2023, in Brazil and its regions, and to estimate the clinical consequences of untreated caries in 2023. A probabilistic cluster sample obtained from the three surveys was analyzed. The oral examinations for caries followed the recommendations of the World Health Organization. High caries experience was identified using the Significant Caries Index (SiC). Clinical consequences of untreated caries were measured using the PUFA index. A hypothesis test was conducted to verify differences in the means of DMFT and components, SiC, DMFT=0, and decayed teeth ≥ 1 among the surveys of 2003, 2010, and 2023. The analysis of data from 34,529 (2003), 7,328 (2010), and 6,704 (2023) adolescents revealed a significant reduction in DMFT from 2.47 (2003) and 2.07 (2010) to 1.67 (2023), and for the SiC group the reduction was from 6.30 (2010) to 5.90 (2023). A significant increase in the prevalence of caries-free was observed, from 34.64% (2003) and 43.49% (2010) to 49.88% (2023), and a significant reduction in the prevalence of adolescents with untreated caries from 48.47% (2003) to 36.85% (2023). Adolescents had on average 0.14 teeth with clinical consequences of untreated caries, with pulp involvement being the most frequent (80.02%), and 8.71% had PUFA≥1. Although 12-year-old adolescents showed a decrease in caries experience, the SiC group, untreated caries, and its clinical consequences represent sociodemographic inequities that need to be addressed.

## Introduction

Dental caries is considered one of the most prevalent non-communicable chronic diseases worldwide, although it is preventable^1^ with well-established determinants.^2^ Its high prevalence also reflects social and economic inequalities, as well as inadequacy and insufficiency of investments in preventive measures and treatment, particularly in low- and middle-income countries.^1^ However, globally, epidemiological studies have shown a trend of decreasing caries prevalence in recent decades, especially in wealthy countries and among 12-year-old adolescents.^3-5^

In Latin America, a decrease in the experience, prevalence, and severity of caries has also been observed among children and adolescents aged 11 to 13, in studies using the World Health Organization (WHO) criteria,^6^ especially in Brazil.^7^ However, this decrease has been marked by geographic and socioeconomic inequality, with a higher disease burden observed among adolescents from lower income households, living in municipalities without treated and fluoridated water, and with worse economic indicators.^8^ It is important to emphasize that caries is determined by a complex interaction of individual and contextual factors, involving biological, behavioral, living conditions, and access to prevention methods and health services.^2,9-11^

The importance of epidemiological surveys for monitoring the oral health of the population, particularly at the age of 12, is worth highlighting, because this age is considered for global monitoring, international comparisons, and tracking disease trends. Dental caries has been evaluated using the criteria recommended by WHO^12^ through the DMFT index (decayed, missing, and filled teeth), which is a globally used index for studying caries distribution in populations.^1^ Given the unequal pattern of caries distribution in populations, other indices have been proposed and used to complement and overcome some of the limitations of the DMFT.^13^ The Significant Caries Index (SiC) is calculated from the DMFT and helps identify the population group with the highest caries experience.^14^ Monitoring untreated caries and its clinical consequences^15^ has also been recommended.^16^

In this context, it is important to highlight the impacts of untreated caries,^17^ as caries lesions can cause pain and discomfort, affecting school performance and the quality of life in children and adolescents.^18,19^ A recent study developed projections showing that untreated caries in permanent teeth is a condition likely to increase over the next 30 years, primarily affecting the elderly population.^20^ Thus, designing and implementing strategies for controlling dental caries, considering the context of the inequalities, particularly among the younger population, becomes extremely relevant and urgent.

In Brazil, the monitoring of oral health indicators is systematically conducted through national oral health surveys,^21-23^ aiming to produce relevant information for the planning, implementation, and reorientation of the National Oral Health Policy (*Política Nacional de Saúde Bucal* – PNSB).^24^ In 2023, the assessment of untreated caries and its clinical consequences was incorporated into the national oral health survey, allowing the attainment of estimates of the severity of untreated caries and associated pathologies.^23^ Therefore, this study aimed to compare caries experience and the prevalence of caries-free adolescents and those with untreated caries among 2003, 2010, and 2023 in Brazil and its regions, and estimate the clinical consequences of untreated caries in 2023.

## Methods

The study was conducted using data from the national oral health surveys of 2003, 2010, and 2023, coordinated by the Brazilian Ministry of Health, including 12-year-old adolescents.^21-23^

### Study population

In 2003, the survey was conducted in 250 municipalities (50 in each of the five Brazilian macroregions). The selection of participants was conducted using a probabilistic cluster sampling method, obtained through three stages of selection: municipalities (primary units), census tracts (secondary units), and blocks and households (tertiary units).^21^ The sampling plan for SB Brasil 2010 consisted of 32 geographical scopes: 27 scopes from state capitals and five scopes from municipalities in the interior of the five Brazilian regions. Participants were selected by cluster sampling in two stages for state capital municipalities and in three stages for municipalities in the interior of the regions.^22^ In 2023, the study population consisted of individuals residing in households in urban areas across the entire national territory. The distribution of the population by strata and demographic scopes, the sample size for state capitals and the interior, and the expected sampling errors for DMFT estimates are detailed in the technical project of the survey.^23^ For the 12-year-old index age group, the sample was obtained through a single-stage process, meaning that all households in the selected census tracts were surveyed for potential participants. This methodological choice was based on the fact that 12-year-old residents are rare, making the random selection of households inefficient, so all households in the selected tracts were included in the sample.^23^ In all three surveys, the sampling plan allowed for the generation of estimates for the five Brazilian regions, whose data will be analyzed in this article.

### Data Collection

In all three surveys, data collection was conducted by professionals working in the public health service affiliated with the Unified Health System (SUS), including the examiner (dentist) and an annotator (oral health technician or assistant). Interviews and oral examinations of participants were conducted in their own homes. The data collection instruments had socioeconomic and demographic information, as well as questions related to oral health. The examinations and epidemiological indices used in this study followed the recommendations of WHO. Field teams were trained to perform the stages of data collection in households. ^21-23^ In 2023, all recommended biosafety measures were implemented to ensure the safety of participants and field teams, addressing the particular challenges of the COVID-19 pandemic.^23^ Examiners received theoretical and practical training on the codes and criteria for assessing oral health conditions, achieving substantial or almost-perfect agreement coefficients (simple Kappa or weighted Kappa > 0.65 in 2003 and 2010, and > 0.61 in 2023).^25,26^

In 2003 and 2010, in-person training and calibration workshops lasting at least 32 hours were conducted. In 2010, trainings on trauma and fluorosis were conducted using the in-lux method, where photographs of oral conditions simulated scenarios examiners might encounter during fieldwork.^27^ In 2023, due to the COVID-19 pandemic, theoretical and practical training for field teams was conducted online through synchronous and asynchronous activities in a virtual learning environment built on the Moodle® platform. The theoretical training lasted a minimum of 16 hours, with activities introducing the methodology, oral health conditions, and their codes and criteria. This was followed by training using the in-lux method with photographs.^26^ Calibration covered the following oral health conditions: deciduous dentition (crown caries, pufa, and occlusal condition) and permanent dentition (crown caries, PUFA, DAI, and dental trauma).^23^ The methods used were detailed in previous publications.^21-23,26,27^

### Dental Caries

Dental caries was assessed based on the WHO codes and criteria in all three surveys (12). From this assessment, the DMFT index (number of permanent decayed, missing, and filled teeth) was calculated. The mean DMFT and its components—decayed, filled with caries, filled without caries, and missing teeth—were obtained. Based on the index analysis, the prevalence of participants free of caries, i.e., those without any caries experience (DMFT = 0), was also estimated. Additionally, the total number of decayed teeth was calculated by the sum of decayed teeth and filled teeth with caries. From this count, it was possible to estimate the prevalence of participants with one or more teeth with untreated caries (number of decayed teeth ≥ 1). The third of the sample with the highest caries experience was identified using the Significant Caries Index (SiC).^14^ This index is calculated as follows: individuals are ranked according to their DMFT values; the top third of the population with the highest caries rates is selected, and the mean DMFT of this subgroup is calculated.

The clinical consequences of untreated caries lesions were assessed only in the 2023 survey,^23^ in permanent teeth with decayed crowns, using the PUFA index to estimate the severity of caries and associated pathologies (pulp involvement, ulceration, fistula, and dentoalveolar abscess), complementing the information obtained through the DMFT index.^15^ The mean PUFA index, the mean and proportion of its components relative to the index, and the PUFA prevalence (i.e., the percentage of participants with one or more clinical consequences of untreated caries, PUFA≥1) were presented.

### Data Analysis

The sample was characterized according to region, sex, and race/skin color in 2003, 2010, and 2023, and according to level of education only in 2010 and 2023, as this variable was not included in the 2003 national survey. Self-reported race/skin color was classified following the methodology of the Brazilian Institute of Geography and Statistics: White, Black, Asian, Mixed-race, and Indigenous. The level of education was assessed based on the highest grade (level) completed by the participant, converted into years of schooling with good performance (no grade retention), and categorized as 0 (did not attend school), 1–4, 5–8, 9–11, and 12 or more years of education. For data analysis, given the complexity of the sample, estimates of means, percentages, their respective standard errors and 95% confidence intervals were calculated using the ‘svy’ command in Stata v. 18 (StataCorp LP, College Station, USA). This command considers the sampling design variables and applies weights derived from the sampling process. The sampling weights for the 2003 survey were calculated by Queiroz et al.^28^ The observed change in the mean DMFT and its components, the SiC group’s DMFT, and the prevalence of adolescents caries-free or with at least one tooth with untreated caries among surveys were calculated, comparing 2023 to 2010 and 2003. This change was expressed as Δ2023_2010, Δ2023_2003, and Δ2010_2003. The significance of the change was tested using a hypothesis test, dividing Δ by the standard error of the change, and estimating the confidence interval of the change at a 5% significance level. The null hypothesis was rejected when the confidence interval did not include zero. Differences in mean values across the years were estimated using the ‘lincom’ command in Stata®.

### Ethical Aspects

All SBBrasil surveys were approved by research ethics committees and adhered to the guidelines of Resolution No. 466/2012 of the Brazilian National Health Council, ensuring the adoption of the Informed Consent Form to participants or their legal guardians.

## Results

The sample consisted of 34,529, 7,328, and 6,704 12-year-old adolescents in 2003, 2010, and 2023, respectively. A balanced gender distribution was observed in all three surveys, with approximately half of the participants being male (50.63%, 49.47%, and 50.96%) and female (49.37%, 50.53%, and 49.04%). Regarding race/skin color, most participants identified as Mixed-race (45.90%, 42.72%, and 48.17%) or White (41.27%, 43.23%, and 40.20%). Most participants had 5 to 8 years of schooling in 2010 (82.43%) and 2023 (97.35%), and the percentage of 12-year-olds who had never attended school was low (0.34% and 0.29%) (Table 1).

**Table 1:** Percentage of 12-year-old participants according to sociodemographic variables in the years 2003, 2010, and 2023, in Brazil and its regions.

Variables	2003		2010		2023	
	n	w% (95%CI)	n	w% (95%CI)	n	w% (95%CI)
Brazil	34,529		7,328		6,704	
Region						
North	6,208	9.16 (6.89; 12.09)	1,743	9.32 (7.94; 10.92)	1,627	10.85 (9.01; 13.02)
Northeast	7,322	31.97 (27.99; 36.23)	2,041	12.16 (10.57; 13.96)	2,522	29.16 (25.35; 33.29)
Southeast	8,052	38.52 (34.55; 42.64)	1,342	60.07 (55.06; 64.86)	686	38.30 (33.37.43.47)
South	7,098	13.51 (11.74; 15.49)	1,010	11.66 (9.99.13.57)	863	13.47 (11.16.16.18)
Central-West	5,849	6.83 (5.91; 7.89)	1,192	6.78 (5.82; 7.89)	1,006	8.22 (6.425.10.46)
Sex	34,529		7,328		6,703	
Male	16,349	50.63 (50.24.51.02)	3,639	49.47 (46.39; 52.56)	3,391	50.96 (47.2; 54.71)
Female	18,180	49.37 (48.98; 49.76)	3,689	50.53 (47.44; 53.61)	3,312	49.04 (45.29; 52.80)
Race/Skin Color	34,253		7,328		6,597	
White	14,750	41.27 (39.34; 43.23)	2,897	43.23 (41.04; 45.44)	2,132	40.20 (36.43; 44.08)
Black	3,120	9.96 (9.19; 10.79)	712	11.58 (8.64; 15.33)	782	10.60 (8.04.13.85)
Asian/Indigenous	1,220	2.86 (2.59; 3.16)	206	2.48 (1.90; 3.23)	94	1.02 (0.68; 1.53)
Mixed race	15,163	45.90 (43.66; 48.16)	3,513	42.72 (39.80; 45.69)	3,589	48.17 (44.17; 52.20)
Level of Education*	-	-	7,303		6,561	
Did not go to school	-	-	19	0.34 (0.14; 0.79)	38	0.29 (0.14; 0.58)
1 to 4 years of education	-	-	1,079	12.41 (10.85; 14.15)	253	2.35 (1.67; 3.30)
5 to 8 years of education	-	-	5,801	82.43 (80.44; 84.27)	6,270	97.35 (96.39; 98.06)
9 to 11 years of education	-	-	336	4.30 (3.11; 5.91)	0	0.00
≥12 years of education	-	-	68	0.51 (0.27; 0.93)	0	0.00

*This variable was not collected in the 2023 survey.

The mean DMFT index obtained in each survey, along with its components (permanent decayed, filled with caries, filled without caries, and missing teeth) and the deltas for comparisons among the surveys, are presented in Table 2. The mean DMFT index was 2.47 in 2003, 2.07 in 2010, and 1.67 in 2023. From 2003 to 2023, the mean index decreased by nearly one dental element (-0.80) in Brazil. A significant reduction in the DMFT index was observed in 2023 compared to 2003 and 2010, both for Brazil as a whole and for the Northeast, Southeast, and South regions. The South region showed the largest reductions in the DMFT index between 2023 and 2003 (-1.23) and between 2023 and 2010 (-1.06). Between 2003 and 2010, significant changes in the DMFT index were only observed in the Central-West region; no changes were detected in 2023 compared to previous years (Table 2).

**Table 2:** Mean DMFT and number of decayed, missing, and filled teeth at the age of 12 in Brazil and per region, and variation among the years 2023, 2010, and 2003.

Variable	DMFT (95% CI)			Differences among surveys		
	2003	2010	2023	Δ2010-2003	Δ2023-2003	Δ2023-2010
Brazil	2.47 (2.33; 2.62)	2.07 (1.84; 2.29)	1.67 (1.49; 1.86)	**-0.41 (-0.68; -0.14)**	**-0.80 (-1.04; -0.56)**	**-0.39 (-0.68; -0.10)**
North	3.07 (2.83; 3.32)	3.16 (2.76; 3.56)	2.73 (2.31; 3.14)	0.08 (-0.38; 0.55)	-0.35 (-0.82; 0.13)	-0.43 (-1.01; 0.14)
Northeast	2.84 (2.43; 3.26)	2.63 (2.14; 3.12)	1.84 (1.50; 2.18)	-0.22 (-0.86; 0.42)	**-1.01 (-1.54; -0.47)**	**-0.79 (-1.38; -0.19)**
Southeast	1.99 (1.92; 2.05)	1.72 (1.41; 2.03)	1.25 (0.94; 1.56)	-0.26 (-0.58; 0.05)	**-0.74 (-1.06; -0.42)**	-0.47 (-0.91; -0.04)
South	2.23 (2.13; 2.33)	2.06 (1.68; 2.43)	1.00 (0.79; 1.21)	-0.17 (-0.55; 0.22)	**-1.23 (-1.46; -1.00)**	-1.06 (-1.49; -0.63)
Central-West	3.18 (2.94; 3.43)	2.63 (2.42; 2.84)	2.80 (1.80; 3.79)	-0.55 (-0.88; -0.23)	-0.39 (-1.42; 0.63)	0.16 (-0.85; 1.18)
Decayed						
Brazil	1.53 (1.37; 1.69)	1.21 (1.03; 1.37)	1.04 (0.91; 1.16)	-0.31 (-0.54; -0.09)	**-0.49 (-0.69; -0.28)**	-0.17 (-0.37; 0.02)
North	2.26 (2.01; 2.51)	2.27 (1.89; 2.65)	1.81 (1.44; 2.17)	0.01 (-0.44; 0.46)	**-0.45 (-0.89; -0.01)**	-0.46 (-0.99; 0.07)
Northeast	2.11 (1.67; 2.56)	1.91 (1.52; 2.30)	1.26 (1.03; 1.49)	-0.21 (-0.79; 0.38)	-0.86 (-0.79; 0.38)	-0.65 (-1.10; -0.20)
Southeast	0.95 (0.91; 0.99)	0.85 (0.64; 1.05)	0.74 (0.52; 0.97)	-0.11 (-0.32; 0.10)	-0.21 (-0.44; 0.02)	-0.11 (-0.44; 0.02)
South	1.12 (1.07; 1.17)	1.26 (0.95; 1.56)	0.51 (0.33; 0.69)	0.13 (-0.18; 0.45)	**-0.61 (-0.80; -0.43)**	-0.75 (-1.10; -0.39)
Central-West	1.84 (1.74; 1.94)	1.68 (1.44; 1.91)	1.46 (0.98; 1.93)	-0.16 (-0.42; 0.09)	-0.38 (-0.87; 0.10)	-0.22 (-0.75; 0.31)
Missing						
Brazil	0.16 (0.15; 0.17)	0.12 (0.06; 0.19)	0.07 (0.05; 0.09)	-0.04 (-0.10; 0.03)	**-0.09 (-0.11; -0.06)**	-0.05 (-0.11; 0.02)
North	0.39 (0.34; 0.45)	0.24 (0.19; 0.28)	0.19 (0.09; 0.28)	-0.16 (-0.23; -0.08)	**-0.21 (-0.31; -0.10)**	-0.05 (-0.15; 0.05)
Northeast	0.24 (0.23; 0.26)	0.22 (0.10; 0.34)	0.09 (0.06; 0.13)	-0.03 (-0.15; 0.09)	**-0.15 (-0.19; -0.11)**	-0.12 (-0.25; -0.01)
Southeast	0.06 (0.05; 0.06)	***	0.05 (0.02; 0.07)	***	-0.02 (-0.04; 0.01)	***
South	0.11 (0.10; 0.11)	0.04 (0.02; 0.07)	0.02 (0.001; 0.05)	-0.06 (-0.09; 0.04)	**-0.08 (-0.11; -0.06)**	-0.02 (-0.05; 0.01)
Central-West	0.11 (0.10; 0.12)	0.09 (0.06; 0.13)	0.07 (0.02; 0.13)	-0.02 (-0.06; 0.02)	-0.04 (-0.09; 0.02)	-0.02 (-0.08; 0.05)
Filled						
Brazil	0.79 (0.74; 0.83)	0.73 (0.64; 0.83)	0.57 (0.46; 0.67)	-0.06 (-0.16; 0.04)	**-0.22 (-0.34; -0.11)**	-0.17 (-0.31; -0.02)
North	0.43 (0.35; 0.51)	0.65 (0.54; 0.76)	0.73 (0.44; 1.02)	0.22 (0.09; 0.36)	**0.31 (0.01; 0.60)**	0.08 (-0.23; 0.39)
Northeast	0.49 (0.41; 0.56)	0.50 (0.42; 0.58)	0.49 (0.26; 0.72)	0.01 (-0.10; 0.12)	0.001 (-0.23; 0.24)	-0.01 (-0.25; 0.23)
Southeast	0.97 (0.91; 1.03)	0.77 (0.61; 0.93)	0.46 (0.32; 0.61)	**-0.20 (-0.37; -0.03)**	**-0.51 (-0.67; -0.36)**	**-0.31 (-0.52; -0.10)**
South	1.00 (0.91; 1.09)	0.76 (0.58; 0.93)	0.47 (0.34; 0.59)	**-0.24 (-0.44; -0.04)**	**-0.53 (-0.69; -0.38)**	**-0.29 (-0.51; -0.07)**
Central-West	1.23 (1.07; 1.40)	0.87 (0.74; 0.99)	1.27 (0.73; 1.80)	**-0.36 (-0.57; -0.16)**	0.03 (-0.53; 0.59)	0.40 (-0.15; 0.95)

The values in bold represent significant changes during the period, as determined by the hypothesis test; * Estimate with a lower limit of the confidence interval with a negative value.

Analyzing the components of the DMFT index separately, a significant reduction in the mean number of decayed teeth was observed in Brazil between 2003 and 2023. Regionally, a significant reduction in the mean number of decayed teeth in 2023 compared to 2003 and 2010 was noted only in the South. The North and Northeast regions also showed reductions in the mean number of decayed teeth, but these occurred during different periods. On the other hand, the Southeast and Central-West regions showed no reductions across the three surveys. Regarding missing teeth, a significant reduction in the mean number was observed in 2023 compared to 2003 and 2010 only in the Northeast. A significant reduction in the mean number of filled teeth was also observed in 2023 compared to 2003 and 2010, in Brazil and in the Southeast and South regions (Table 2).

**Figure 1:**
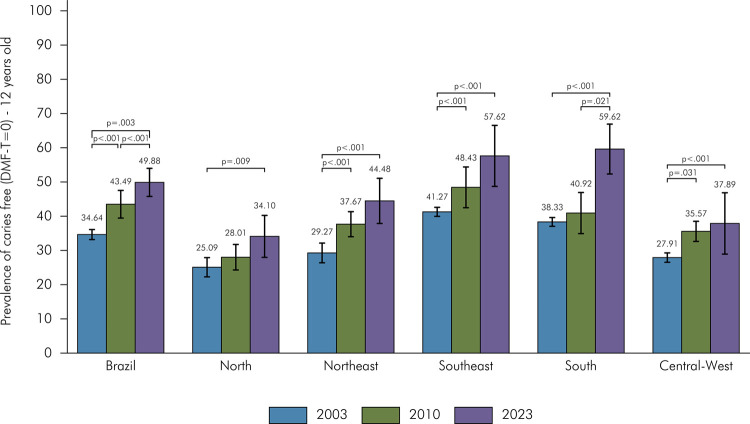
Prevalence of 12-year-old adolescents free of caries (DMFT=0) in Brazil and regions.

*Note: The horizontal bars indicate statistically significant differences in comparisons among the surveys of 2023, 2010, and 2003.

In 2023, a significant increase in the prevalence of 12-year-old adolescents free of caries (DMFT = 0) was observed in Brazil compared to previous surveys (2023: 49.88%; 2010: 43.49%; 2003: 34.64%). However, notable regional differences were identified, with the highest proportions of caries-free adolescents found in the South (59.62%) and Southeast (57.62%) regions, and the lowest proportions in the North (34.10%) and Central-West (37.89%) regions. A significant increase in the percentage of caries-free adolescents was observed in all regions when comparing 2023 to 2003 (Figure 1).

**Figure 2:**
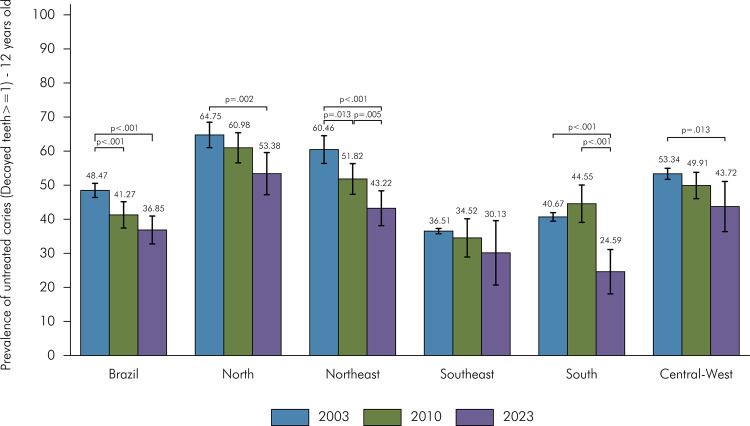
Prevalence of 12-year-old adolescents with one or more teeth with untreated caries (Decayed teeth > = 1) in Brazil and regions.

*Note: The horizontal bars indicate statistically significant differences in comparisons among the surveys of 2023, 2010, and 2003.

In 2023, there was a significant reduction in the prevalence of 12-year-old adolescents with at least one tooth with untreated caries in Brazil compared to the 2003 survey (2023: 36.85%; 2003: 48.47%). This same trend was observed across all regions except for the Southeast, where no significant changes were observed over the years. The Northeast region was the only region to show a significant reduction in the prevalence of untreated caries in 2023 (43.22%) compared to 2010 (51.82%) and 2003 (60.46%). The South region also had a reduction between 2010 (44.35%) and 2023 (24.59%). Regional differences were identified in 2023, with the South (24.59%) and Southeast (30.13%) having the lowest proportions of 12-year-old adolescents with at least one tooth with untreated caries, while the North (53.38%) and Central-West (43.72%) regions had the highest proportions (Figure 2).

In Brazil, when evaluating the DMFT index of 12-year-old adolescents in the SiC group, a significant decrease was observed between the 2023 and 2010 surveys, a trend also seen in the Northeast region. In the South, significant reductions were noted between 2023 and 2003, as well as between 2023 and 2010, with the lowest SiC in 2023 (4.75) compared to other regions. In the Southeast, a significant increase in SiC was observed between the 2010 and 2003 surveys, followed by a reduction between 2023 and 2010. In the North and Central-West, no changes were observed in the SiC group’s DMFT across the three surveys, with these regions having the highest values compared to the other regions in 2023 (6.72; 7.26) (Table 3).

**Table 3:** Mean DMFT of 12-year-old adolescents in the SiC group in Brazil, per region, and variation between the years 2023, 2010, and 2003.

Variable	DMFT SiC Group (95% CI)			Differences among surveys		
	2003	2010	2023	Δ2010-2003	Δ2023-2003	Δ2023-2010
Brazil	6.15 (5.85; 6.46)	6.30 (6.11; 6.48)	5.90 (5.56; 6.25)	0.14 (-0.21; 0.50)	-0.24 (-0.70; 0.21)	**-0.39 (-0.78; 0.00)**
North	6.32 (6.12; 6.51)	6.64 (6.12; 7.16)	6.72 (6.21; 7.22)	0.32 (-0.23; 0.87)	0.39 (-0.14; 0.94)	0.07 (-0.64; 0.80)
Northeast	6.60 (5.79; 7.42)	6.90 (6.31; 7.48)	5.78 (5.39; 6.18)	0.29 (-0.70; 1.29)	-0.81 (-1.72; 0.09)	**-1.11 (-1.81; -0.40)**
Southeast	5.64 (5.59; 5.74)	6.07 (5.80; 6.34)	5.23 (4.65; 5.80)	**0.42 (0.14; 0.71)**	-0.41 (-0.99; 0.17)	**-0.84 (-1.47; -0.20)**
South	5.83 (5.79; 5.86)	5.79 (5.34; 6.25)	4.75 (4.41; 5.10)	-0.03 (-0.48; 0.42)	**-1.07 (-1.41; -0.72)**	**-1.04 (-1.61; -0.46)**
Central-West	6.43 (6.14; 6.71)	6.63 (6.26; 7.00)	7.26 (6.15; 8.37)	0.20 (-0.26; 0.67)	0.83 (-0.31; 1.98)	0.62 (-0.54; 1.79)

The values in bold represent significant changes during the period, as determined by the hypothesis test.

Regarding the PUFA index, each 12-year-old adolescent in Brazil had on average 0.14 teeth with clinical consequences of untreated caries. The highest average was observed in adolescents in the North (0.20), Central-West (0.19), and Northeast (0.16) regions. The most frequent clinical consequence among 12-year-old adolescents in Brazil was pulp involvement, representing 80.02% of the PUFA index. The PUFA prevalence was 8.71% in Brazil, with the highest percentages found in the Central-West (13.43%) and North (12.88%) regions (Table 4).

**Table 4:** Mean PUFA index, mean and proportion of index components, and percentage (with 95% confidence interval) of 12-year-old adolescents with one or more clinical consequences of untreated caries (PUFA ≥ 1) in Brazil and per region, in the year 2023.

Scope: Brazil and regions	n	PUFA	Pulp involvement		Ulcer		Fistula		Abscess		PUFA≥1		
		Mean*	Mean*	%*	Mean*	%*	Mean*	%*	Mean*	%*	%*	CI95%	
												L.I.	L.S.
Brazil	6,698	0.14	0.11	80.02	0.02	10.92	0.01	6.61	0.00	2.44	8.71	7.24	10.18
North	1,626	0.20	0.16	78.42	0.03	12.59	0.01	5.34	0.01	3.65	12.88	9.94	15.83
Northeast	2,52	0.16	0.13	79.06	0.02	12.58	0.01	5.95	0.00	2.40	10.14	7.62	12.66
Southeast	686	0.11	0.10	86.42	0.01	4.83	0.01	8.02	0.00	0.73	6.58	3.84	9.31
South	862	0.08	0.05	61.16	0.02	22.91	0.01	7.57	0.01	8.36	5.43	3.04	7.81
Central-West	1,004	0.19	0.15	80.81	0.02	11.90	0.01	5.87	0.00	1.42	13.43	6.95	19.92

*Weighted estimates with post-stratification adjustment for age, sex, and education.

## Discussion

Brazilian adolescents aged 12 presented, on average, fewer than two permanent teeth with caries experience in 2023, along with a higher proportion of caries-free, reflecting a positive result compared to the national oral health surveys of 2003 and 2010. However, regional inequalities still persist. The same decreasing trend was observed for the SiC group, but only when comparing 2023 and 2010. The decrease in caries experience was not consistent across all regions, and reductions in the mean components of the DMFT index also occurred unevenly among the surveys. Another important result highlighting disparities in Brazil in 2023 is the DMFT for adolescents with the highest caries experience, which is approximately 3.5 times greater than the national average.

The results also revealed a significant reduction in the prevalence of 12-year-olds with untreated caries only when comparing the results for Brazil from 2023 and 2003, with regional differences. This indicates a challenge to be addressed in this age group, especially considering the clinical consequences (PUFA), which were studied for the first time in a population-based survey in the country. These consequences can affect adolescents’ well-being, health, and school performance.^1,19^

The reduction observed in the DMFT reinforces the decreasing trend already discussed in other studies.^3-5,7,8^ However, while this result is positive and possibly reflects progress in public oral health policies implemented in Brazil in recent decades,^8,24^ it also highlights inequalities, as the decrease is not occurring uniformly across all regions and still reveals the issue of high caries experience and untreated caries. Regarding caries-free adolescents, this increase – while significant over the years – did not reach 50%, a percentage that is still modest compared to Germany, for example, which had 78.8% of 12-year-olds caries-free in 2016.^29^

Adolescents with high caries experience have DMFT from 2.8 (SiC = 4.75) to 4.3 (SiC = 7.26) times higher than the average of the population studied (DMFT = 1.67), as seen in other studies conducted in Brazil^30,31^ and other countries.^13,29^ A SiC lower than 3 in 12-year-olds was recommended as a global target for 2015.^14^ These results reinforce the importance of identifying SiC groups so that disease control strategies and resources can be better targeted and optimized for those who carry the greatest burden of disease.

Regarding untreated caries (number of decayed teeth ≥1), two regions stood out when comparing surveys. The Northeast region was the only one to show a significant reduction in prevalence across the three surveys, while no significant change was observed over time in the Southeast region. However, in 2023, the prevalence of adolescents with at least one tooth with untreated caries was 43.22% in the Northeast and 30.13% in the Southeast. Other marked differences were also identified among the remaining regions of the country, as found by Freire et al.^8^ in 2010, although the calculation was made in relation to the DMFT index (DMFT≥1) and not solely based on decayed teeth.

Untreated caries has been considered one of the greatest public health challenges globally and has gained prominence in international literature in recent years,^5,32^ due to its negative impacts and the compromises it causes in people’s lives,^1^ especially in children and adolescents.^18,19,33^ A significant part of this challenge lies in inequities, as the disease disproportionately affects socially vulnerable populations^30,34^ that are often not benefited by intervention strategies and policies, potentially exacerbating existing inequalities.^35^ In this study, regional inequalities were also observed in relation to untreated caries and the PUFA prevalence, highlighting the need to ponder populational disease control strategies considering equity-promoting approaches, given the complex range of determinants of dental caries.^2,9,10,36^

The information on the mean value (0.14) and prevalence of PUFA (8.71%) obtained in this study was valuable for understanding the clinical consequences of untreated caries among 12-year-old Brazilian adolescents, enabling comparisons with other population-based studies in different contexts, and provided insights for planning new strategies to address untreated caries. A study conducted in the North region of Brazil with 12-year-old school adolescents (n = 376) examined the clinical consequences of untreated dental caries, individual characteristics, and environmental factors in self-reported oral health measures, revealing a pufa/PUFA mean of 0.3 (± 0.7) and a PUFA prevalence of 13.8%,^33^ which are higher than the values found for Brazil and the North region in this study. On the other hand, a study conducted in Nigeria, including school adolescents aged 13 to 16 years (n = 347), showed lower values compared to the present study: a PUFA mean value of 0.05 (± 0.2) and a PUFA prevalence of 2.9%.^37^

Pulp involvement was the most prevalent clinical consequence among adolescents (80.02% of the PUFA index). This clinical condition is particularly concerning due to its severity, especially considering the age of the participants. Its management is not simple and may require comprehensive and intersectoral oral health service management processes, since it involves planning strategies to reach this population, in addition to resources and investments in primary care, and points to the expansion and strengthening of specialized care. It is important to emphasize that, even within a public, universal healthcare system organized into different levels of care like the Brazilian SUS), socioeconomic inequalities in oral health persist, affecting adolescents from more vulnerable social strata.^8,10,38^

Addressing and controlling dental caries involves tackling the multiple determinants of the health-disease process,^1,35^ with a focus on preventive and promoting measures for oral health, as well as intersectoral actions.^36,39^ These considerations align with the WHO recommendations outlined in the ‘Global Strategy and Action Plan on Oral Health 2023–2030’, which contains a set of actions that include strengthening policies, health promotion, prevention, and education actions, as well as ensuring access to oral health services.^40^

This study highlighted the potential of the epidemiological surveys conducted in Brazil to guide targeted actions for improving access to dental services and strengthening health promotion strategies by integrating oral health into public policy agendas.^35,40^ The SiC index identified groups with high caries experience, while the PUFA index was used to assess clinical consequences of untreated caries and thus overcome the limitations of DMFT in measuring caries severity. However, an in-depth analysis of the conditions studied and the indicators related to social inequalities in Brazil and its regions proved to be relevant and necessary.

## Final Considerations

Dental caries is still a public health problem for the Brazilian population, following an international trend. This study showed that there was a significant reduction in the experience of caries and an increased prevalence of 12-year-old adolescents free of caries when comparing the three national surveys. There was also a significant reduction in the prevalence of adolescents with high caries experience (between 2023 and 2010) and with untreated caries (between 2023 and 2003). These outcomes may be associated with the impact of the National Oral Health Policy implemented in recent decades, which includes the use of fluoridated toothpaste, preventive programs, and particularly the introduction of oral health teams within the Family Health Strategy. However, striking regional disparities persisted, and untreated caries and its clinical consequences remain challenges to be addressed. Beyond highlighting sociodemographic inequities, these issues also impose a biological, social, and financial burden on the healthcare system. Preventive measures, oral health promotion, intersectoral actions, and strategies to expand access to both primary and specialized care need to be strengthened to ensure health and well-being in this young population.
